# Formulation and *In Vitro* Characterisation of Withaferin A-Loaded Liposomal Gels for the Topical Management of Chronic Inflammatory Skin Conditions

**DOI:** 10.3389/bjbs.2025.14847

**Published:** 2025-12-01

**Authors:** Mandeep Kaur Marwah, Hala Shokr, Karan Singh Rana, Yukta Sameer Hindalekar, Rosie Kainth, Parmida Babaei, Shakil Ahmad

**Affiliations:** 1 Aston Medical School, College of Health and Life Sciences, Aston University, Birmingham, United Kingdom; 2 Pharmacy Division, School of Health Sciences, Faculty of Biology, Medicine and Health, The University of Manchester, Manchester, United Kingdom; 3 School of Biosciences, College of Health and Life Sciences, Aston University, Birmingham, United Kingdom

**Keywords:** liposomes, topical-delivery, gels, inflammation, drug-targetting

## Abstract

Chronic inflammatory skin conditions such as psoriasis, eczema, and acne are driven by sustained inflammation, oxidative stress, and impaired tissue repair. Current treatments often lead to adverse effects with prolonged use highlighting the need for safer, targeted alternatives. Withaferin-A, a bioactive compound derived from *Withania somnifera*, has demonstrated potent anti-inflammatory and antioxidant properties in various disease models. This study explored the potential of Withaferin-A liposome-loaded gels for topical delivery, testing their efficacy in an inflamed skin model. Withaferin-A liposomes were prepared using the ethanol injection method and incorporated into hydroxypropyl methylcellulose (HPMC) gels. *In vitro* release studies using a permeable insert system were used to compare release profiles of Withaferin-A from liposomal gels. Cytotoxicity was assessed via XTT assay on human umbilical vein endothelial cells (HUVEC) and human dermal fibroblasts (HDFa). Inflammation was induced with tumour necrosis factor-alpha (TNF-α), and anti-inflammatory effects measured using enzyme-linked immunosorbent assays for interleukin-6 (IL-6) and matrix metalloproteinase-9 (MMP9). Reactive oxygen species (ROS) levels were quantified using the DCFDA assay. Cytotoxicity studies using the XTT assay on HUVEC and HDFa cells revealed good biocompatibility at lower Withaferin-A concentrations (0–1 µM), though reduced viability was observed at 5 µM. *In vitro* release studies revealed sustained release of Withaferin-A from liposomal gels, with significantly slower release over 6 h compared to solution at 99.53% ± 3.47% and 48.87% ± 4.51% respectively. Anti-inflammatory effects were evaluated following TNF-α-induced inflammation, with Withaferin-A loaded gels significantly reducing IL-6 secretion in a dose-dependent manner in both HUVECs (38.90 ± 5.34 to 19.15 ± 3.56 pg/mL) and HDFa cells (40.05 ± 2.23 to 10.42 ± 2.02 pg/mL). Withaferin-A treatment also reduced ROS levels and lowered MMP-9 secretion in HDFa cells from 408.80 ± 13.05 pg/mL to 195.00 ± 7.55 pg/mL, indicating potential for tissue remodelling. In summary, WA-loaded liposomal gels demonstrated effective anti-inflammatory activity and sustained drug release while maintaining biocompatibility at therapeutic concentrations. These findings support their potential as a novel strategy for managing inflammatory skin diseases by improving drug bioavailability and promoting tissue repair.

## Introduction

Chronic inflammatory skin conditions, such as psoriasis, eczema, and acne, are characterised by persistent inflammation, oxidative stress, and tissue damage [1, 2]. These disorders not only significantly impact patients’ quality of life but also pose ongoing clinical challenges due to their recurrent flare-ups and the need for sustained management [3]. While corticosteroids remain a cornerstone of the treatment, their use is frequently accompanied by local side effects like skin thinning, which can exacerbate long-term skin damage as well as potential systemic complications with prolonged or widespread application [4, 5]. This highlights the need for safer, more effective alternatives that can target inflammation and oxidative stress more precisely, without the undesirable consequences of traditional treatment regimens.

Pro-inflammatory cytokines such as Interleukin 6 (IL-6) and enzymes such as Matrix Metallopeptidase 9 (MMP-9) are frequently overexpressed in chronic skin conditions, contributing to prolonged inflammation and impaired tissue repair [6]. Elevated IL-6 perpetuates the inflammatory response, while increased MMP-9 activity disrupts collagen synthesis, hindering structural integrity and delaying the healing process [7]. Furthermore, inflammation-associated overproduction of Reactive Oxygen Species (ROS) damages dermal cells and impairs fibroblast function, further exacerbating tissue injury [8]. This cycle of inflammation and ROS disrupts skin health, making it essential to target and regulate these factors to restore balance, promote tissue regeneration, and improve healing in chronic skin conditions [9].

Withaferin A, a bioactive steroidal lactone derived from *Withania somnifera* (Ashwagandha), has demonstrated potent anti-inflammatory and antioxidant properties in various disease models [10]. Its therapeutic potential is particularly notable in conditions where inflammation and oxidative stress are key drivers of pathogenesis. For instance in endothelial cells exposed to palmitic acid, Withaferin A significantly reduced ROS production and pro-inflammatory cytokine release by inhibiting the Inhibitor of kappa B kinase subunit beta/Nuclear Factor kappa B (IKKβ/NF-κB) pathway, thereby restoring insulin signalling and nitric oxide production [11]. Similarly, in high-fat diet–induced obese mice, oral administration of Withaferin A improved hepatic insulin sensitivity and glucose metabolism by enhancing antioxidant enzyme activity and downregulating inflammatory mediators such as NF-κB, Tumor Necrosis Factor-alpha (TNF-α), and Cyclooxygenase-2 (COX-2) [12]. Despite its pharmacological promise, the application of Withaferin A in dermatological contexts remains limited due to poor aqueous solubility, chemical instability, and insufficient skin permeability—factors that collectively hinder its topical efficacy [13].

To address these challenges, we propose the use of liposomes—nano-sized lipid vesicles—as a delivery system for Withaferin A. Liposomes can encapsulate hydrophobic compounds like Withaferin A, thereby protecting them from degradation, enhancing solubility, and enabling sustained controlled release. A recent *ex vivo* study demonstrated that liposome-loaded gels enhanced the solubility and stability of ADT-OH, a hydrophobic compound, promoted dermal retention, and enabled sustained release while preserving its biological activity in murine skin. Furthermore, liposomes loaded with naringenin suspended in polymer-based gels have demonstrated the ability to penetrate dermal cells and significantly retard drug release, supporting their suitability for the controlled dermal delivery of antioxidant and anti-inflammatory agents. The ability of liposomes to cross the skin barrier, especially when formulated for dermal delivery, makes them an ideal method for delivering Withaferin A to the site of action in inflammatory skin conditions [14].

In addition to liposomes, we propose the use of aqueous gels as a vehicle for topical liposome delivery. Gels provide a biocompatible and stable matrix that supports extended release and improved local absorption [15–17]. When use in combination with liposomes, gels offer enhanced skin penetration, improving the bioavailability and effectiveness of Withaferin A at the target site [14, 18]. This combination of liposomes and gels is designed to address the limitations of traditional topical formulations, offering a more targeted and efficient approach to managing inflammation and oxidative stress in chronic skin disorders.

By incorporating liposomes and gels in the delivery of Withaferin A, we aim to overcome the challenges associated with its topical application. This strategy not only enhances the therapeutic potential of Withaferin A but also provides a safer, more controlled means of managing inflammation and oxidative stress, both of which play central roles in the pathogenesis of chronic inflammatory skin conditions.

## Methods

### Materials

Phosphatidylcholine (PC) (catalogue #1535733), Withaferin A (catalogue #W4394), hydroxypropyl methylcellulose (HPMC) (catalogue #09963), polyethylene glycol (grade ≥ 99.5%, catalogue #W294004), cholesterol (grade ≥ 99%, catalogue #C8667) and Tween 20 (grade ≥ 95%, catalogue #P1379) were obtained from Sigma-Aldrich (Dorset, England). All other reagents including ethanol and phosphate-buffered saline were obtained from Fisher Scientific, Loughborough, England. Ultrapure water was obtained from a Milli-Q purification system (Millipore, Billerica, MA, US). Polycarbonate filter, pore size 400 nm, 200 nm, 100 nm, and 50 nm, were obtained from Sigma-Aldrich. Tumour necrosis factor-alpha (TNF-α) (Cat. No. 210-TA-005) was purchased from R&D Systems, Abingdon, UK.

### Cell Culture and Treatment

Primary Human Umbilical Vein Endothelial cells (HUVEC, PromoCell, Stourbridge, England, catalogue. #C-12203) were cultured in full growth media (EGM-2) (PromoCell, Cat. # C-22211) supplemented with Fetal Calf Serum 0.02 mg/mL, Epidermal Growth Factor 5 ng/mL, Basic Fibroblast Growth Factor 10 ng/mL, Insulin-like Growth Factor 20 ng/mL, Vascular Endothelial Growth Factor 0.5 ng/mL, Ascorbic Acid 1 μg/mL, Heparin 22.5 μg/mL, Hydrocortisone 0.2 μg/mL (supplement kit, Promocell, Cat. # C-39211) and 5 mL of 1X Penicilin/Streptomycin (Lonza, Slough, England, Cat. # LZDE17-602E). Cells were maintained at 37 °C in a humidified incubator with 5% CO_2_, with media refreshed every 48 h. Sub-culturing was performed at 70%–80% confluency, and cells were used up to passage 5 for experiments.

Human dermal fibroblasts (HDFa; ThermoFisher, Cat. #C0135C), isolated from adult skin, were cultured using Human Fibroblast Expansion Basal Medium (ThermoFisher, Cheshire, England, Cat. #M106500) supplemented with the low serum growth supplement (LSGS) kit (ThermoFisher Cat. #S00310), which includes 2% v/v fetal bovine serum, 1 μg/mL hydrocortisone, 10 ng/mL human epidermal growth factor, 3 ng/mL basic fibroblast growth factor, and 10 μg/mL heparin.

### Cellular Toxicity of Withaferin A Towards HUVEC and HDFa Cells

To evaluate the cytotoxicity of Withaferin A on HUVEC and HDFa cells, a 2,3-bis-(2-methoxy-4-nitro-5-sulphophenyl)-2H-tetrazolium-5-carboxanilide (XTT) assay was conducted to assess cell viability following 24-hour drug exposure. Cells were seeded at a density of 50 × 10^3^ cells per well in 96-well plates and cultured for 2 days. After this period, the culture medium was removed and replaced with 100 µL of media containing Withaferin A at concentrations ranging from 0.1 to 5 µM. Cells were incubated for a further 24 h at 37 °C. Following treatment, 25 µL of XTT/menadione reagent (12.5:1 ratio) was added to each well and incubated for 3 h at 37 °C. Absorbance was then measured at 450 nm, with higher absorbance values indicating greater cell viability. The cytotoxic effect of Withaferin A was determined by examining the decrease in absorbance as drug concentration increased.

### Liposome Preparation

Liposomes were prepared by the ethanol injection method established by Batzri and Korn, 1973 [19]. Based on Withaferin A cytotoxicity data, two non-cytotoxic concentrations were selected for further evaluation: 0.1 µM and 0.5 µM final concentrations at the cellular level. To achieve these working concentrations following a final 1:20 dilution upon application to cells (as per previous protocol [14]), Withaferin A was incorporated into liposomes at 1 mg/mL and 5 mg/mL, referred to as WA-L and WA-H, respectively. These loading amounts were calculated by accounting for the entrapment efficiency of Withaferin A.

Phosphatidylcholine and cholesterol were dissolved in ethanol at a molar ratio of 16:8 μM, along with the appropriate amount of Withaferin A. To enhance skin permeation, 10% w/w Tween 20 (relative to total lipid content) was added during the lipid mixing stage as described previously [15]. The organic phase was injected into 1 mL of PBS using a syringe pump under continuous magnetic stirring at 25 °C (above lipid phase transition temperature). The lipid suspension was stirred for 5 min at room temperature. The subsequent multilamellar vesicles were extruded through 400 nm, 200 nm, and 100 nm polycarbonate membranes (Avanti Mini Extruder) to produce unilamellar vesicles. Unentrapped Withaferin A and ethanol were removed by dialysis against distilled water over 24 h using Slide-A-Lyzer dialysis cassettes. Mean particle size, polydispersity index (PDI) and surface charge (zeta potential) were measured using established protocols previously validated in our laboratory [20].

### Determination of Liposome Entrapment Efficiency

The entrapment efficiency of Withaferin A in liposomes was determined by comparing the drug concentration in liposome samples before and after dialysis. To disrupt the liposome bilayer, 9 parts acetonitrile were added to 1 part of the liposome formulation. The mixture was then centrifuged at 16,000 RCF, and the concentration in the supernatant was assayed. UV analysis was performed using a Spark® plate reader (TECAN, Switzerland) to determine the encapsulation efficiency of Withaferin A in the liposomal formulations (Equation 1):

$$E=\left(D_{t}-D_{s}\right)\;/D_{t}\times 100$$ (1)

where *E* refers to encapsulation efficiency (%), *D*_*t*_ refers to total drug content (mg), and *D*_*s*_ refers to drug content in supernatant (mg).

### Withaferin A Loaded Aqueous Gel Formulation

Hydroxypropyl methylcellulose (HPMC) gels were initially prepared at 10% w/v in distilled and deionised water and mixed overnight using a mechanical homogeniser (Polytron PT 3100 D) at 3000 rpm. These gels were subsequently diluted 1:1 with both WA-L and WA-H liposomal formulations. The final formulations consisted of 5% w/v HPMC gels. These concentrations were selected to ensure that, when the gels are applied to cells at a 1:1 dilution, the maximum possible release would not exceed the target final Withaferin A concentrations used in cytocompatibility assays. All gel formulations contained 15% v/v ethanol.

### *In Vitro* Diffusion of Withaferin A From Liposomal Gel Formulations

To investigate the release of Withaferin A from liposomes incorporated into polymer gels, a permeable insert model system was employed as described previously [15]. This system was designed to compare release profiles of Withaferin A solution, gel or WA-H loaded liposomes. To facilitate comparison, the concentration of Withaferin-A in solution and gel formulations was matched to the final concentration achievable from the higher-dose liposomal gel only. This ensured any differences observed in release profiles were attributable to the formulation matrix rather than dose-dependent effects. A 4 cm^2^ cylindrical cell culture Thincert™ insert (400 µm pore size) was filled with 1 mL of formulation and placed into a 6-well Thincert™ plate. The release into 4 mL of PBS from both solution and liposomal gels was quantified. The plates were maintained at 35 °C on a shaking platform, and 0.5 mL samples were collected over 6 h with volume replacement, followed by analysis using UV spectroscopy.

### Assessment of TNF-α Toxicity to Define a Non-Cytotoxic Inflammatory Model

To explore the potential of Withaferin A in attenuating inflammation induced by TNF-α, we first conducted a dose-response assay using HUVEC and HDFa’s. Cells were exposed to increasing concentrations of TNF-α (0, 1, 5, 10, and 20 ng/mL) for 3 h, after which the media was replaced with fresh culture medium and cells were incubated for an additional 24 h to determine the optimal concentration that would not induce cytotoxicity Based on cell viability analysis, 5 ng/mL TNF-α for 3 h was selected for subsequent experiments, as it consistently induced an inflammatory response while maintaining cell viability.

### Investigating the Anti-Inflammatory and Matrix Remodelling Effects of Withaferin A Formulation in HUVEC and HDFa Cells

To assess the anti-inflammatory and matrix remodelling regulatory potential of Withaferin A, levels of IL-6 and MMP9 were quantified in cell culture supernatants using the Human DuoSet enzyme-linked immunosorbent assay (ELISA) (R&D Systems, Minneapolis, USA, DY202 and DY911 respectively). HUVEC and HDFa’s were seeded into 12-well plates and allowed to adhere overnight. Cells were then treated with TNF-α (5 ng/mL) for 3 h, after which the media was replaced with fresh culture medium and cells were incubated for an additional 24 h to induce inflammation. Following this, cells were washed with PBS and incubated with WA-L and WA-H gels for 24 h. Afterwards, media was collected and stored at − 80 °C until the assay was performed following the manufacturer’s instructions.

### Measurement of Intracellular Reactive Oxygen Species (ROS) Levels in HUVEC and HDFa’s

Intracellular reactive oxygen species (ROS) levels were measured using the DCFDA/H2DCFDA Cellular ROS Assay Kit (Abcam, Cambridge, England ab113851), following the manufacturer’s protocol with minor modifications. HUVEC and HDFa’s were seeded into black-walled, clear-bottom 96-well plates at a density of 1 × 10^4^ cells/well and allowed to adhere overnight under standard culture conditions. Cells were then subjected to experimental treatments (e.g., TNF-α stimulation followed by ± Withaferin A) as per the study design. Following incubation, cells were gently washed with 1X PBS and then incubated with 100 µL of 25 µM DCFDA solution (prepared in serum-free medium) for 45 min at 37 °C in the dark. After staining, intracellular ROS levels were quantified by measuring fluorescence at excitation/emission wavelengths of 485/535 nm using a Spark® plate reader (TECAN, Switzerland). Fluorescence readings were normalised to untreated controls, and results were expressed as relative ROS levels. All measurements were performed in triplicate wells, and data are presented as mean ± SEM from at least three independent experiments.

### Statistical Tests

Unless otherwise specified, all data are expressed as mean ± standard deviation (SD). Each experiment included a minimum of five independent replicates (n = 3). The exact sample size for each study is indicated in the corresponding figure legend, with equal group sizes maintained throughout. Data distribution was first verified using the Shapiro–Wilk test. Statistical significance between two groups was assessed using a t-test, while comparisons among more than two groups were evaluated using one-way analysis of variance (ANOVA), followed by Tukey’s *post hoc* multiple comparison test where applicable. All statistical analyses were performed using GraphPad Prism version 10.2.0 (GraphPad Inc., La Jolla, CA, USA).

## Results

### Cellular Toxicity of Withaferin A Towards HUVEC and HDFa’s

**FIGURE 1:**
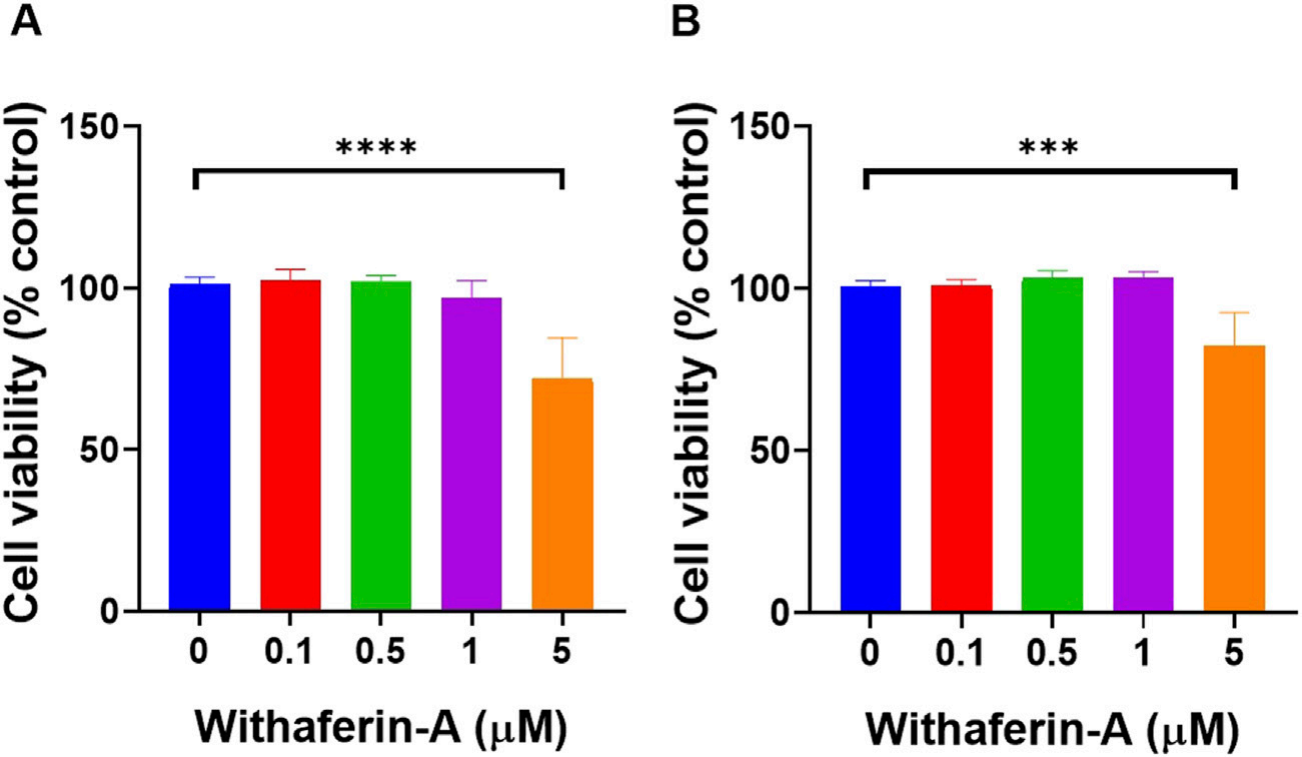
Withaferin A concentrations up to 5 µM did not significantly affect cell viability on both **(A)** HUVEC and **(B)** HDFa’s. Cytotoxicity was assessed following exposure of cells to increasing concentrations of Withaferin A. Cells were seeded at 50 × 10^3^ cells per well in 96-well plates and incubated for 2 days before exposure to the drug. Following 24-hour incubation with Withaferin A, cell viability was measured using the XTT assay. Data are presented as mean ± standard deviation, n = 4 independent experiments. Statistical significance was assessed by One-way ANOVA: ***p < 0.001, ****p < 0.0001.

To ensure that Withaferin A could be evaluated for its anti-inflammatory effects without inducing cytotoxicity, a dose-dependent viability assessment was conducted in HUVEC and HDFa cells (Figure 1). Identifying a concentration that preserves cell viability is critical to distinguish true anti-inflammatory activity from effects caused by cellular stress or damage. HUVEC cell viability (Figure 1A) showed no significant change at lower concentrations, with the average viability at 0 µM being 101.2% ± 2.183% and at 0.1 µM being 102.5% ± 3.375%. At 0.5 µM, viability was 102% ± 1.782%, and at 1 μM, it slightly decreased to 96.98% ± 5.292%. However, the most notable reduction in viability occurred at 5 μM, with the average viability dropping to 71.98% ± 12.59%. Statistical analysis revealed a significant decrease in viability between the 0 μM and 5 µM concentrations (p < 0.0001), indicating that the treatment significantly reduced HUVEC viability at the highest dose tested.

Similarly, HDFa cell viability (Figure 1B) was assessed with results showing minimal changes in viability at lower concentrations, with the average viability at 0 µM being 100.7% ± 1.873%, at 0.1 µM, it was 100.8% ± 2.06%,at 0.5 µM being 103.3% ± 2.24%, and at 1 μM, it was 103.5% ± 1.828%. However, a significant reduction in viability was observed at 5 μM, where the viability dropped to 82.33% ± 10.24%. Statistical analysis revealed a significant decrease in viability between the 0 μM and 5 µM concentrations (p < 0.001) indicating that the treatment significantly reduced HDFa viability at the highest concentration tested.

### Characterisation of Withaferin A Loaded Liposomal Formulations

Liposomes are effective drug carriers with key properties such as size, zeta potential, polydispersity index, and entrapment efficiency playing a critical role in their performance. Evaluation of the physicochemical consistency of these formulations aims to elucidate the influence of Withaferin A concentration on liposomal characteristics and assess their suitability for pharmaceutical development and therapeutic application.

Liposome formulations were characterised at two concentrations of Withaferin A (1 mg/mL and 5 mg/mL, WA-L and WA-H respectively), both of which demonstrated comparable physicochemical properties (Table 1). The mean particle size was 121.7 ± 6.99 nm for the 1 mg/mL (WA-L) formulation and 117.3 ± 5.81 nm for the 5 mg/mL (WA-H) formulation. The polydispersity index was also comparable, with values of 0.16 ± 0.05 and 0.15 ± 0.04 for the two concentrations, respectively. The zeta potential for WA-L had a slight negative value of −0.33 ± 2.86 mV, while WA-H showed a slight positive value of 0.48 ± 1.58 mV. Finally, entrapment efficiency was similar for both formulations, with 72.56% ± 5.33% for WA-L and 70.4% ± 3.46% for WA-H. ANOVA Statistical analysis revealed no significant differences between the two liposome groups across all parameters (p > 0.05), indicating formulation characteristics were consistent regardless of the Withaferin A concentration.

**TABLE 1:** Physicochemical properties of liposomal formulations containing Withaferin A at concentrations of 1 mg/mL and 5 mg/mL.

Parameter	1 mg/mL Withaferin A	5 mg/mL Withaferin A
Liposome size (nm)	121.7 ± 6.987	117.3 ± 5.809
Polydispersity index (PD)	0.158 ± 0.0507	0.152 ± 0.04438
Zeta potential (ZP) (mV)	−0.326 ± 2.863	0.48 ± 1.581
Entrapment efficiency (%)	72.56 ± 5.335	70.4 ± 3.467

### *In Vitro* Diffusion of Withaferin A From Liposomal Gel Formulations

**FIGURE 2:**
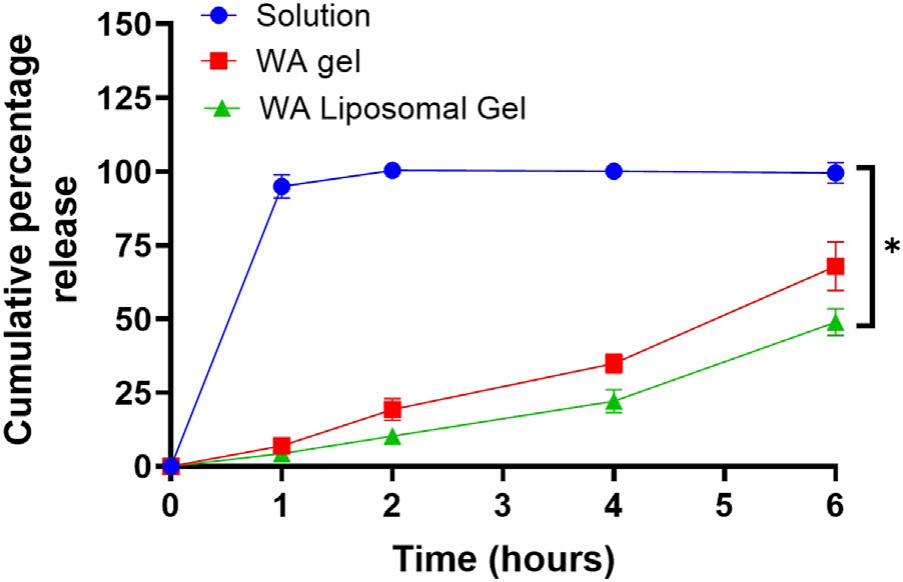
Liposomal gels sustain the release of Withaferin A (WA) more effectively than solution and gel formulations over 6 h. *In vitro* release was assessed using a permeable insert system, held at 35 °C. The concentration of Withaferin A in the solution and gel formulations was matched to the final achievable concentration from the higher-dose liposomal gel (5 mg/mL) to ensure comparability. Release was quantified by UV spectrophotometry. Data are presented as mean ± standard deviation, n = 3 independent experiments. Statistical significance was assessed by One-way ANOVA: *p < 0.05.

To investigate the controlled release potential of Withaferin A from different formulations, diffusion studies were conducted over 6 h using a permeable insert model. The cumulative release of Withaferin A into PBS was measured for three groups: Withaferin A solution, gel, and liposomal gel (Figure 2). To specifically assess the impact of formulation type rather than drug concentration, only the highest loading of Withaferin A liposomes was used to prepare the gel, with concentrations across the other groups matched accordingly. Rapid release was observed from the Withaferin A solution, with 94.9% ± 4.0% released after 1 h and over 99% by 2 h, indicating immediate availability of the compound in solution form. In contrast, the Withaferin A gel showed a more sustained release profile, with 6.97% ± 1.72% released at 1 h, 19.33% ± 3.69% at 2 h, and reaching 67.9% ± 8.31% by 6 h. The Withaferin A liposomal gel exhibited the slowest and most controlled release, with 4.33% ± 1.53% released at 1 h, 10.3% ± 2.0% at 2 h, and 48.87% ± 4.51% at 6 h. This formulation demonstrated a gradual release profile, likely due to the combined barrier effects of both the gel matrix and liposomal encapsulation. Comparing the release profiles across the 6-h time point, there was a statistically significant difference (p < 0.05) between all three formulations, confirming the ability of gel-based and liposomal systems to modulate and sustain the release of Withaferin A effectively.

### Assessment of TNF-α Toxicity to Define a Non-Cytotoxic Inflammatory Model

To develop a reliable *in vitro* model of skin inflammation that mimics inflammatory conditions without inducing cytotoxicity, it was essential to first determine a TNF-α concentration that stimulates inflammatory signalling while maintaining cell viability. To achieve this, a dose-response assessment of TNF-α toxicity was conducted in both HUVEC and HDFa’s using the XTT viability assay.

**FIGURE 3:**
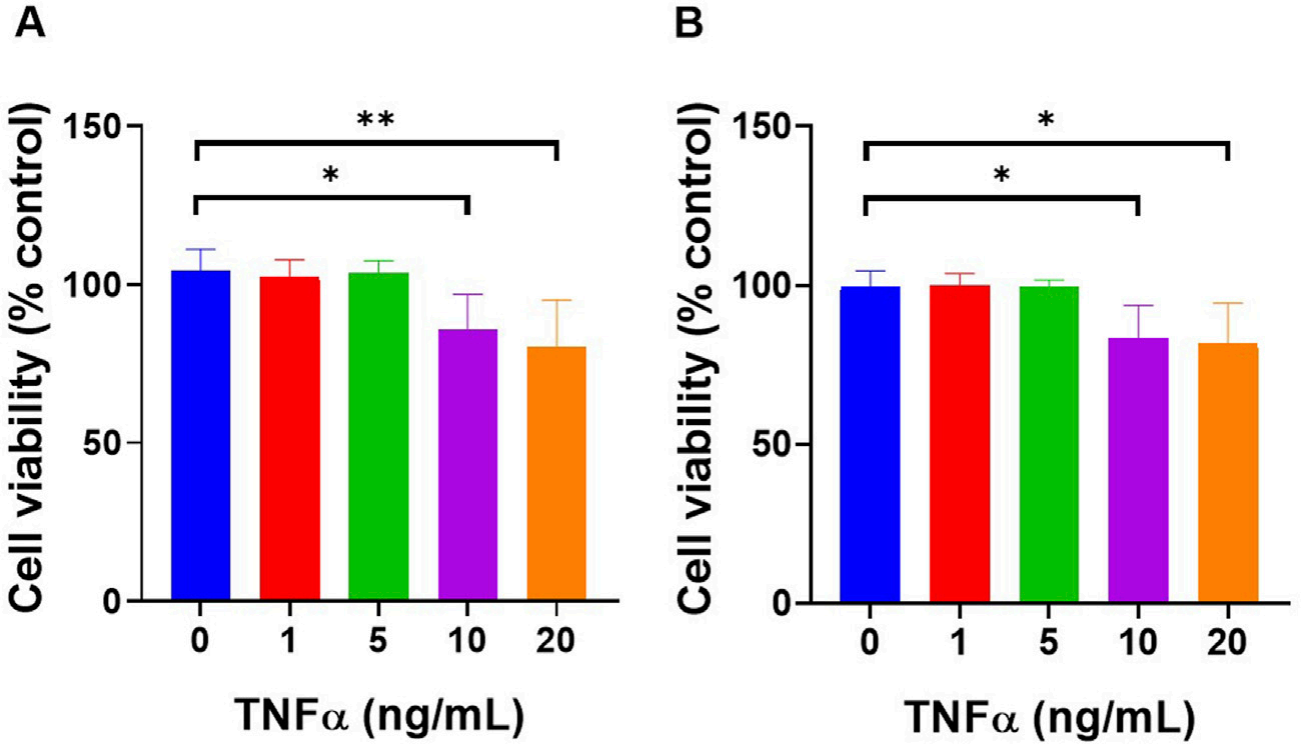
TNF-α concentrations up to 5 ng/mL did not significantly affect cell viability on both **(A)** HUVEC and **(B)** HDFa’s when exposed for 3 h. Cells were treated with increasing concentrations of TNF-α (0, 1, 5, 10, and 20 ng/mL) for 3 h, followed by 24 h of incubation with fresh culture medium before performing the XTT assay. Data are presented as mean ± standard deviation, n = 3 independent experiments. *p < 0.05, **p < 0.01, as determined by One-way ANOVA.

TNF-α toxicity in HUVEC cells was assessed using the XTT assay following exposure to varying concentrations of treatment (Figure 3A). At 0 ng/mL, cell viability was 104.5% ± 6.56%, at 1 ng/mL, it was 102.3% ± 5.443% and at 5 ng/mL, the viability remained stable at 103.4% ± 4.072%. A significant reduction in cell viability was observed at 10 ng/mL, with viability decreasing to 85.53% ± 11.35%, and the most significant reduction was observed at 20 ng/mL, where the viability dropped to 80.32% ± 14.67%. Statistical analysis revealed a significant decrease in cell viability between 0 ng/mL and 10 ng/mL (p < 0.05), and between 0 ng/mL and 20 ng/mL (p < 0.01).

Similarly, TNF-α toxicity in HDFa’s was assessed (Figure 3B). At 0 ng/mL, the cell viability was 99.69% ± 4.921%, at 1 ng/mL, it was 99.84% ± 3.813%, and at 5 ng/mL, the viability remained similar at 99.65% ± 1.996%. A significant reduction in cell viability was observed at 10 ng/mL, with viability decreasing to 83.39% ± 10.34%, and at 20 ng/mL, it dropped further to 81.54% ± 12.82%. Statistical analysis revealed a significant decrease in cell viability between 0 ng/mL and 10 ng/mL as well as between 0 ng/mL and 20 ng/mL (p < 0.05), indicating TNF-α toxicity significantly increased at higher concentrations.

### Investigating Anti-inflammatory and Matrix Remodelling Effects of Withaferin A in HUVEC and HDFa’s

Following TNF-α stimulation (5 ng/mL, 3 h) and a subsequent 24-hour incubation period, cells were treated with WA-L and WA-H liposomal gel formulations. IL-6 levels were quantified in both HUVECs and HDFa’s, given its central role in endothelial activation and dermal inflammation and its frequent elevation in inflammatory skin conditions. MMP-9 levels were evaluated in HDFa’s due to its involvement in extracellular matrix degradation and tissue remodelling, commonly dysregulated in chronic inflammatory dermal pathologies.

**FIGURE 4:**
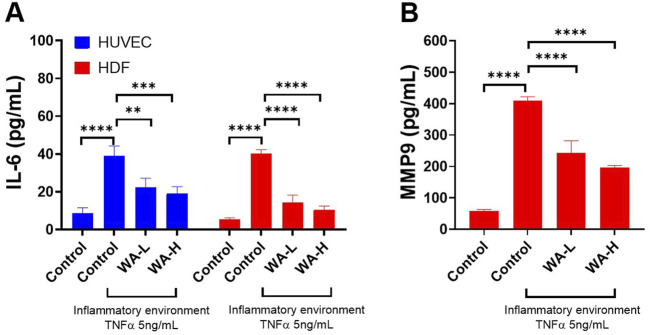
Withaferin A (WA) Reduces TNF-α-Induced Inflammation and Matrix Metalloproteinase levels in HUVEC and HDFa’s. **(A)** IL-6 secretion in HUVECs and HDFa’s and treatment with Withaferin A-loaded liposomes (either 1 mg/mL noted as WA-L or 5 mg/mL noted as WA-H), resulted in a concentration-dependent reduction of IL-6 levels. **(B)** MMP-9 secretion in HDFa’s was significantly elevated by TNF-α stimulation however treatment with Withaferin A-loaded liposomal gel reduced MMP-9 levels. Cells were stimulated with 5 ng/mL TNF-α for 3 h and treated with Withaferin A-loaded liposomal gels (WA-L and WA-H corresponding to a Withaferin A loading to 1 and 5 mg/mL) for 24 h followed by appropriate ELISA analysis. Data are presented as mean ± standard deviation, n = 3 independent experiments. Statistical significance was assessed by One-way ANOVA: **p < 0.01, ***p < 0.001, ****p < 0.0001.

TNF-α significantly increased IL-6 levels in HUVECs compared to the untreated control group (38.90 ± 5.34 pg/mL vs. 8.50 ± 3.08 pg/mL, p < 0.0001) (Figure 4A). Treatment with Withaferin A-loaded liposomal gel led to a concentration-dependent reduction in IL-6 secretion, with levels of 22.36 ± 4.80 pg/mL, p < 0.01) and 19.15 ± 3.56 pg/mL, p < 0.001) observed for WA-L and WA-H liposomal gel, respectively, compared to the TNF-only group. These results suggest that Withaferin A formulation significantly attenuates TNF-α-induced inflammatory signalling in a dose dependant manner in HUVECs.

A similar trend was observed in the HDFa cells (Figure 4A). A significant increase in IL-6 levels was observed in the TNF-α-treated group compared to untreated controls (40.05 ± 2.23 pg/mL vs. 5.49 ± 0.74 pg/mL, p < 0.0001). Treatment with Withaferin A-loaded liposomal gel markedly reduced IL-6 secretion to 14.32 ± 3.93 pg/mL and 10.42 ± 2.02 pg/mL for WA-L and WA-H formulations respectively, both showing significant reductions compared to TNF-α alone p < 0.0001). These findings indicate strong anti-inflammatory potential of Withaferin A liposomal gel formulations in dermal fibroblasts.

MMP-9 secretion in HDFa’s was significantly elevated following TNF-α stimulation (Figure 4B). Compared to the untreated control group (58.00 ± 5.48 pg/mL), TNF-α treatment resulted in a marked increase in MMP-9 levels (408.80 ± 13.05 pg/mL, p < 0.0001). Treatment with Withaferin A-loaded liposomal gel significantly reduced MMP-9 secretion to 243.00 ± 38.43 pg/mL and 195.00 ± 7.55 pg/mL for WA-L and WA-H formulations respectively (p < 0.0001), indicating potent inhibitory effects of the formulation on inflammatory matrix metalloproteinase expression.

### Measurement of Intracellular Reactive Oxygen Species (ROS) Levels in HUVEC and HDFa’s

**FIGURE 5:**
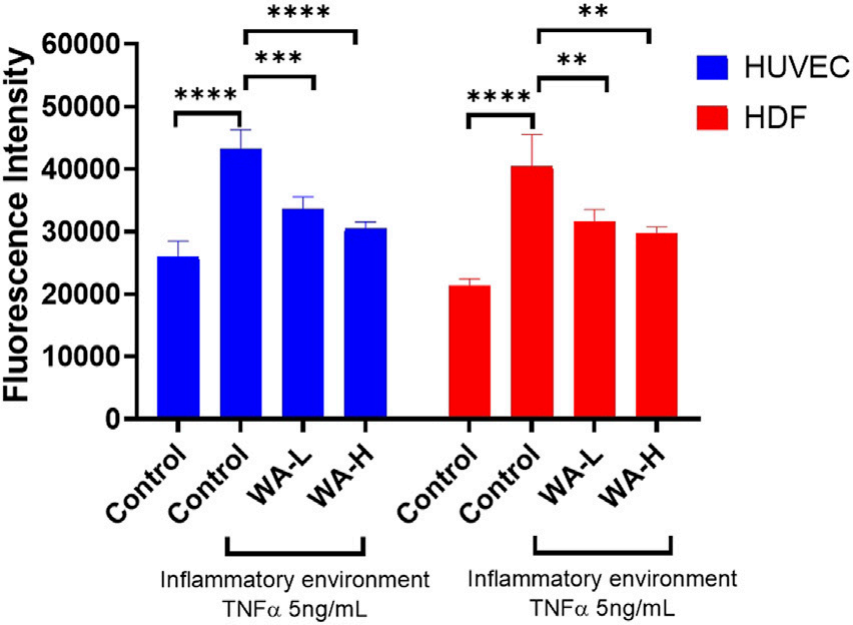
Withaferin A Reduces TNF-α-Induced Oxidative Stress in HUVEC and HDFa’s in a Dose-Dependent Manner. Intracellular ROS levels were measured in HUVEC and HDFa cells following 4-hour TNF-α stimulation (5 ng/mL) and subsequent treatment with Withaferin A liposomal gel (WA-L and WA-H corresponding to a Withaferin A loading to 1 and 5 mg/mL) for 24 h followed by quantification using DCFDA Cellular ROS Assay Kit. Results are expressed as mean ± standard deviation and analysed by One-way ANOVA. n = 3 independent experiments. **p < 0.01, ***p < 0.001, ****p < 0.0001.

To evaluate effect of Withaferin A on oxidative stress in HUVEC’s induced by TNF-α (5 ng/mL, 3 h), intracellular ROS levels were quantified using the DCFDA Cellular ROS Assay Kit (Figure 5). Cells treated with TNF-α showed a significant increase in ROS production (43,315 ± 2,945 AU) compared to untreated controls (25,983 ± 2,519 AU; p < 0.0001). Treatment with WA-L liposomal gel reduced ROS levels to 33,547 ± 1,958 AU, representing a significant decrease compared to the TNF-α-only group (p < 0.001). WA-H liposomal gel further reduced ROS levels to 30,557 ± 1,035 AU, showing an even more pronounced suppression (p < 0.0001) compared to the TNF-α group.

Similarly, the antioxidant effect of Withaferin A in HDFa’s, ROS levels was assessed. Stimulation with TNF-α (5 ng/mL) significantly elevated intracellular ROS levels (40,435 ± 5,137 AU) compared to untreated controls (21,305 ± 1,150 AU; p < 0.0001). Treatment with WA-L liposomal gel reduced ROS levels to 31,631 ± 1,869 AU, showing a significant decrease relative to TNF-α alone (p < 0.01). Similarly, WA-H liposomal gel resulted in further suppression of ROS to 29,747 ± 1,007 AU (p < 0.01). These results demonstrate that Withaferin A significantly reduces TNF-α-induced oxidative stress in both HUVEC and HDFa cells in a dose-dependent manner, supporting its potential as an anti-inflammatory agent in dermal applications.

## Discussion

The development of effective and well-tolerated treatments for chronic inflammatory skin conditions, such as psoriasis, eczema, and acne, remains a significant clinical challenge due to the persistent interplay between inflammation, oxidative stress, and tissue damage. Although, current therapies, including topical corticosteroids, offer symptomatic relief, their long-term use is limited by adverse effects such as skin atrophy, delayed wound healing, and, in some cases, systemic side effects including hypothalamic-pituitary-adrenal axis suppression [4]. These drawbacks underscore the need for safer and more targeted therapeutic alternatives that can effectively modulate inflammatory and oxidative pathways without compromising skin integrity or systemic health.

In this context, Withaferin A—a bioactive steroidal lactone derived from *Withania somnifera* presents a promising alternative due to its well-documented anti-inflammatory, antioxidant, and anti-proliferative properties. Liposomal gel formulations offer additional advantages by enhancing drug stability, improving dermal penetration, and enabling sustained release, thereby increasing therapeutic efficacy while minimising systemic absorption and local irritation.

Characterisation studies of Withaferin A-loaded liposomal formulations demonstrated consistent physicochemical properties across different drug concentrations. Parameters such as liposomes size, polydispersity index, zeta potential, and entrapment efficiency fell within acceptable ranges, confirming formulation suitability for topical application [21]. The incorporation of Tween 20 in the formulation served to enhance lipid bilayer flexibility and facilitate skin permeation [16]. Given its hydrophobic nature Withaferin A is presumed to localise within the lipid bilayer of the liposomes [21, 22]. The lack of change in entrapment or vesicle size with increasing drug concentration implies that bilayer saturation was not reached, indicating additional capacity for drug incorporation [23].

The results of this study support the hypothesis that Withaferin A, when delivered via liposomal formulations, can overcome many of the challenges associated with its topical application. Specifically, liposomes may offer a more effective delivery system by enhancing the controlled release of Withaferin A. *In vitro* release studies demonstrated that the liposomal gel formulations provide a sustained release profile over a 6-h period, which was more prolonged compared to both the solution and gel formulations. This effect can be attributed to the dual barrier properties provided by the liposomal bilayer and the hydrogel matrix which support controlled drug diffusion; a feature critical in managing chronic conditions that require prolonged therapeutic exposure. The contribution of this matrix effect is well-supported by previous studies. For example, liposomal gels containing naringenin demonstrated drug release of up to 25% over 24 h, in contrast to 40%–80% from liposomes alone and up to 100% from solution-based formulations [16]. Similarly, Glavas-Dodov et al. (2002) observed liposomal gel formulations of lidocaine HCl achieved slower release rates than hydrogels containing the drug without liposomes, again highlighting the additive effect of the liposomal and gel matrices in slowing drug diffusion [24]. This controlled release suggests that liposomal formulations could provide a more consistent and prolonged therapeutic effect, which is crucial for managing chronic inflammation and oxidative stress in skin conditions [25]. Future work will include *ex vivo* skin permeation and retention studies to directly quantify the localisation and bioavailability of Withaferin A in the epidermis and dermis, thereby validating the formulation’s ability to overcome skin barrier limitations.

To assess the anti-inflammatory potential of Withaferin A in the context of skin inflammation a non-cytotoxic inflammatory model was established using TNF-α as the pro-inflammatory stimulus. TNF-α plays a central role in the pathogenesis of inflammatory skin disorders by activating endothelial and dermal cells, thereby upregulating downstream mediators such as IL-6 and MMP-9. Dose-response cytotoxicity assays in HUVECs and HDFa confirmed that TNF-α concentrations up to 5 ng/mL did not compromise cell viability, thereby validating the use of this concentration to induce an inflammatory response without confounding cytotoxic effects. Subsequent analyses confirmed that TNF-α stimulation at this concentration led to an increase in IL-6 and MMP-9 expression, indicating successful induction of an inflammatory phenotype. This observation is consistent with previous studies; for example, TNF-α at 5 ng/mL has been shown to induce pro-thrombotic changes in HUVECs without reducing viability [26], and similarly, primary mouse lung fibroblasts exposed to 5 ng/mL TNF-α exhibited increased inflammatory responses while maintaining cell viability [27]**.** These findings collectively support the suitability of this concentration for modelling inflammation *in vitro* and for assessing the therapeutic effects of anti-inflammatory agents such as Withaferin A.

The findings of this study reinforce the hypothesis that Withaferin A, when delivered via liposomal gel formulations, can significantly modulate inflammatory responses and oxidative stress in endothelial and dermal cells. The findings demonstrate the effectiveness of Withaferin A-loaded liposomes in reducing IL-6 secretion in both HUVECs and HDFa’s following TNF-α stimulation. TNF-α at 5 ng/mL significantly increased IL-6 levels in both cell types, consistent with its role in inflammatory skin conditions. Treatment with Withaferin A-loaded liposomal gel led to a concentration-dependent reduction in IL-6 secretion, with the highest reductions observed at liposomes loaded with 5 mg/mL Withaferin A. The findings of this study are further supported by previous research demonstrating the anti-inflammatory and antioxidant potential of Withaferin A in HUVECs. While the current study did not directly investigate molecular signalling pathways, previous work has shown Withaferin A can significantly reduce ROS production and suppress TNF-α and IL-6 by inhibiting the IKKβ/NF-κB signalling pathway [11]. These effects were associated with improved insulin signalling and nitric oxide production, indicating a reversal of inflammation-induced endothelial dysfunction. Such mechanistic insights align with the current observations of reduced IL-6 levels in HUVECs and HDFs following TNF-α stimulation, reinforcing the therapeutic relevance of Withaferin A in conditions characterised by oxidative stress and chronic inflammation.

In addition to its anti-inflammatory effects, Withaferin A also exhibited inhibitory effects on matrix metalloproteinase-9 (MMP-9) expression in HDFa’s. While other MMPs, such as MMP-1 and MMP-3, also contribute to tissue remodelling, this study specifically focused on MMP-9 due to its prominent role in inflammatory skin pathologies. MMP-9 is a zinc-dependent endopeptidase that contributes to extracellular matrix degradation and aberrant tissue remodeling, processes that are dysregulated in chronic inflammatory skin conditions like psoriasis and eczema. TNF-α stimulation led to a significant upregulation of MMP-9 secretion, which was markedly reduced following treatment with Withaferin A-loaded liposomal gel, further emphasising the potential of Withaferin A formulations to modulate tissue remodelling in inflammatory skin disorders. This observation is further supported by previous studies in metastatic cancer models, where Withaferin A significantly inhibited TGF-β-induced MMP-9 expression and activity [28]. The mechanism was attributed to suppression of Akt phosphorylation and downregulation of MMP-9 at both mRNA and protein levels. Taken together, these data suggest that Withaferin A may exert regulatory control over matrix remodelling pathways, highlighting its broader therapeutic potential in inflammatory skin pathologies where extracellular matrix disruption is a hallmark.

Additionally, the antioxidant properties of Withaferin A were evident through a significant reduction in intracellular ROS levels in both HUVEC and HDFa’s. TNF-α-induced ROS generation, a hallmark of oxidative stress in inflammatory environments. Treatment with Withaferin A, particularly at higher concentrations (5 mg/mL loading), significantly reduced ROS. The dose-dependent reduction in ROS suggests the therapeutic potential of Withaferin A as a therapeutic antioxidant agent. As discussed previously, a related study demonstrated that Withaferin A effectively suppressed palmitic acid-induced oxidative stress and inflammation in HUVECs by inhibiting ROS production, downregulating both TNF-α and IL-6 expression [11]. These findings collectively support the dual antioxidant and anti-inflammatory role of Withaferin A, strengthening its potential as a therapeutic candidate for inflammatory skin disorders.

These results are particularly relevant given the central role of both inflammation and oxidative stress in the pathogenesis of chronic inflammatory skin conditions. The ability of Withaferin A-loaded liposomes to both attenuate inflammatory cytokine release and reduce oxidative stress in dermal cells presents a promising strategy for improving therapeutic outcomes in such conditions. Further studies are needed to explore the *in vivo* efficacy of these formulations, particularly in animal models of chronic inflammation and wound healing, to validate their potential for clinical application, including direct comparisons with standard topical anti-inflammatory agents such as corticosteroids to benchmark therapeutic performance. The controlled release profile observed in the *in vitro* studies with liposomal formulations suggests that Withaferin A could provide prolonged anti-inflammatory and antioxidant effects, potentially offering a more effective and sustainable treatment approach compared to conventional therapies.

### Conclusion

The findings from this study underscore the promising therapeutic potential of Withaferin A, particularly when delivered via liposomal gel formulations, for managing chronic inflammatory skin conditions. The liposomal formulations demonstrated favourable physicochemical properties, ensuring effective drug delivery and sustained release, which is critical for the prolonged management of inflammation and oxidative stress. Withaferin A liposomal gel formulations exhibited significant anti-inflammatory effects by reducing IL-6 secretion in both HUVEC and HDFa cells, along with potent inhibition of MMP-9 expression in HDFa’s, highlighting its potential to modulate both inflammation and tissue remodelling. Furthermore, its ability to reduce ROS levels in a dose-dependent manner reinforces its role as an effective antioxidant agent in skin applications. These findings suggest that Withaferin A-loaded liposomal gels could offer an innovative and more effective alternative to traditional therapies for chronic inflammatory skin conditions, potentially improving patient outcomes by addressing both inflammation and oxidative damage.

## Summary Table

### What Is Known About This Subject

- Chronic skin inflammation involves oxidative stress and impaired tissue repair
- Current therapies for psoriasis, eczema, and acne often cause side effects with long-term use
- Withaferin-A is a natural compound with strong anti-inflammatory and antioxidant effects

### What This Paper Adds

- Demonstrates safe and effective *in vitro* topical delivery of Withaferin-A using liposomal gels
- Confirms sustained release of Withaferin-A from gels compared with rapid solution release
- Shows dose-dependent reduction of IL-6, ROS, and MMP-9 following TNF-α induced inflammation
- Provides evidence that Withaferin-A gels support anti-inflammatory action and tissue remodelling

### Concluding Statement

- This work demonstrates *in vitro* that Withaferin-A liposomal gels enable sustained topical drug delivery with potent anti-inflammatory and antioxidant effects, supporting their promise as safer alternatives for managing chronic skin inflammation.

## Data Availability

The raw data supporting the conclusions of this article will be made available by the authors, without undue reservation.
